# On the Food of Children

**Published:** 1848-06

**Authors:** 


*On the Food of Children.*—Dr. Thompson, in his "*Researches on the Food of Animals*," after some remarks on the relative quantities of nutritive matter, in various articles of diet, makes the following judicious observations on the appropriate food of children.

"Milk in some form or other, is the true food of children, and the use of arrow root, or any members of the starch class, where the relation of the nutritive to the calorifiant matter is 1 to 26, instead of being as 1 to 2, by an animal placed in the circumstances of a human infant, is opposed to the principles unfolded in the preceding table. In making this statement, I find that there are certain misapprehensions into which medical men are apt to be led at the first view of the subject. To render it clearer, let us recall to mind what the arrow-root class of diet consists of. Arrow-root and tapioca are prepared by washing the roots of certain plants until all the matter soluble in water is removed. Now, as albumen is soluble in water, this form of nutritive matter must in a great measure be washed away; under this aspect we might view the original root before it was subject to the washing process, to approximate in its composition to that of flour. If the latter substances were washed by repeated additions of water, the nitrogenous or nutritive ingredients would be separated from the starchy or calorifiant elements, being partly soluble in water, and partly mechanically removed. Arrow-root may therefore be considered as flour deprived as much as possible of its nutritive matter. When we administer arrow-root to a child it is equivalent to washing all the nutritive matter out of bread, flour, or oat-meal, and supplying it with starch; or it is the same thing approximately as if we gave it starch; and this is in fact what is done, when children are fed upon what is sold in shops under the title of "Farinaceous Food,"—empirical preparations of which no one can understand the composition without analysis. Of the bad effects produced in children by the use of these most exceptionable mixtures, I have had abundant opportunities of forming an opinion, and I am inclined to infer that many of the irregularities of the bowels, the production of wind, &c. in children, are often attributable to the use of such unnatural species of food. It should be remembered that all starchy food deprived of nutritive matter is of artificial production, and scarcely if ever, exists in nature in an isolated form. The administration of the arrow-root class is therefore only admissible when a sufficient amount of nutritive matter has previously been introduced into the digestive organs, or when it is inadvisable to supply nutrition to the system; as in cases of inflammatory action. In such cases the animal heat must be kept up, and for this purpose, calorifiant food alone is necessary. This treament is equivalent to removing blood from the system, since the wasting of the fibrinous tissues goes on, while an adequate reparation is not sustained by the introduction of nutritive food. A certain amount of muscular sustentation is still, however, effected by the arrow-root diet; since according to the preceding tables, it contains about one-third as much nutritive matter as some wheat flours. The extensive use of *oatmeal*, which is attended with such wholesome consequences among the children of all ranks in Scotland, is, however, an important fact, deserving serious considation, and it appears to me, is strongly corroborative of the principles which I have endeavored to lay down.—*Ib.*

